# Salivary Outer Membrane Vesicles and DNA Methylation of Small Extracellular Vesicles as Biomarkers for Periodontal Status: A Pilot Study

**DOI:** 10.3390/ijms22052423

**Published:** 2021-02-28

**Authors:** Pingping Han, Peter Mark Bartold, Carlos Salomon, Sašo Ivanovski

**Affiliations:** 1 School of Dentistry, Faculty of Health and Behavioural Sciences, The University of Queensland, Brisbane, QLD 4006, Australia; 2 Epigenetics Nanodiagnostic and Therapeutic Group, Center for Oral-Facial Regeneration, Rehabilitation and Reconstruction (COR3), School of Dentistry, Faculty of Health and Behavioural Sciences, The University of Queensland, Brisbane, QLD 4006, Australia; 3 School of Dentistry, The University of Adelaide, Adelaide, SA 5000, Australia; mark.bartold@adelaide.edu.au; 4 Exosome Biology Laboratory, Centre for Clinical Diagnostics, The University of Queensland Centre for Clinical Research, Royal Brisbane and Women’s Hospital, The University of Queensland, Brisbane, QLD 4029, Australia; c.salomongallo@uq.edu.au

**Keywords:** bacterial outer membrane vesicles, global DNA methylation, small extracellular vesicles, periodontitis

## Abstract

Periodontitis is an inflammatory disease, associated with a microbial dysbiosis. Early detection using salivary small extracellular vesicles (sEVs) biomarkers may facilitate timely prevention. sEVs derived from different species (i.e., humans, bacteria) are expected to circulate in saliva. This pilot study recruited 22 participants (seven periodontal healthy, seven gingivitis and eight periodontitis) and salivary sEVs were isolated using the size-exclusion chromatography (SEC) method. The healthy, gingivitis and periodontitis groups were compared in terms of salivary sEVs in the CD9+ sEV subpopulation, Gram-negative bacteria-enriched lipopolysaccharide (LPS+) outer membrane vesicles (OMVs) and global DNA methylation pattern of 5-methylcytosine (5mC), 5-hydroxymethylcytosine (5hmC) and N6-Methyladenosine (m6dA). It was found that LPS+ OMVs, global 5mC methylation and four periodontal pathogens (*T. denticola*, *E. corrodens*, *P. gingivalis* and *F. nucleatum*) that secreted OMVs were significantly increased in periodontitis sEVs compared to those from healthy groups. These differences were more pronounced in sEVs than the whole saliva and were more superior in distinguishing periodontitis than gingivitis, in comparison to healthy patients. Of note, global 5mC hypermethylation in salivary sEVs can distinguish periodontitis patients from both healthy controls and gingivitis patients with high sensitivity and specificity (AUC = 1). The research findings suggest that assessing global sEV methylation may be a useful biomarker for periodontitis.

## 1. Introduction

Periodontitis is a highly prevalent chronic inflammatory disease, affecting up to 50% of the global population [1]. It is associated with microbial dysbiosis and characterised by the destruction of periodontal tissues that may result in tooth loss if left untreated [2]. Current clinical diagnosis relies on a manual instrument (probe) and radiography to determine periodontal status by measuring various parameters, such asbleeding on probing (BOP), plaque index (PI), periodontal pocket depth (PPD) and periodontal bone loss [3]. Early detection and diagnosis of periodontitis would allow timely interventions and appropriate treatments [4]. A non-invasive, biologically based diagnostic technique is yet to be developed and it may improve clinical diagnosis for routine periodontal screening and monitoring of periodontitis patients.

Saliva analysis has been investigated widely as a promising vehicle for periodontitis diagnosis [5,6,7,8], due to being non-invasive, easy to access and harboring a wide range of systemic and local components (i.e., cytokines, extracellular vesicles (EVs), bacteria or bacterial by-products, redox enzymes). Besides the common cytokines, salivary redox, such as stress oxidants and antioxidants, has emerged as a potential biomarker for periodontitis [9,10,11], but does not have the ability to differentiate between the stages of periodontal disease. Salivary small extracellular vesicles (sEVs, also named exosomes), a group of biological nanoparticles produced by virtually every species [12,13], are emerging as potential biomarkers for periodontitis [14,15,16,17,18,19,20], owing to their cargos of nucleic acids (microRNAs, DNAs, etc.), lipids and protein. Most of the current literature has only investigated human-derived CD9/CD63/CD81+ sEVs from biofluids, with little attention given to Gram-negative bacteria-derived outer membrane vesicles (OMVs).

Recent studies have explored the potential of sEVs as diagnostic tools in periodontitis. It has been shown that CD9/CD81-positive salivary sEVs were decreased in periodontitis patients compared to healthy controls [14]. Salivary sEV (exosomal) programmed death-ligand 1 (PD-L1) mRNA was increased (*p* < 0.01) in periodontitis versus non-periodontitis subjects [15]. Moreover, 26 salivary sEV (exosomal) proteins were only detected in severe periodontitis patients and 58 proteins were identified only in the healthy group [19]. Three salivary sEV miRNAs (hsa-miR-140-5p, hsa-miR-146a-5p and hsa-miR-628-5p) were significantly increased in periodontitis patients compared to healthy controls [16]. Furthermore, salivary exosomal hsa-miR-125a-3p (AUC = 1) is a potential biomarker for chronic periodontitis compared to healthy controls [20]. However, it remains unknown whether salivary OMVs and human sEV-associated DNA methylation landscapes can be used as potential biomarkers for periodontitis diagnosis.

There is strong evidence that Gram-negative periodontal pathogens, such as *Tannerella forsythia*, *Porphyromonas gingivalis*, *Treponema denticola*, *Prevotella intermedia*, *Fusobacterium nucleatum*, *Campylobacter rectus*, *Peptostreptococcus anaerobius* and *Eikenella corrodens*, are associated with a microbial dysbiosis that results in periodontal attachment loss and disease progression [21,22]. All of these species secrete outer membrane vesicles (OMVs), nano-sized proteoliposomes enriched with an outer layer of lipopolysaccharide (LPS), which play a vital role in intracellular communication, microbial virulence and host immune response [23]. It has been shown that *P. gingivalis* OMVs facilitate bacterial co-aggregation and influence the bacterial composition of periodontal plaque, which may enhance subgingival biofilm growth [24,25]. Detecting saliva LPS+ OMVs and specific bacterium-derived OMVs is important to understand microbiome–host interaction in periodontal disease.

DNA methylation is a heritable epigenetic change involving the addition of a methyl group to either cytosine or adenine, which is capable of modulating gene expression [26]. The most common form of DNA methylation is 5 methylcytosine (5mC) and 5mC can be demethylated to 5-hydroxymethylcytosine (5hmC) [27]. Beyond 5mC and 5hmC, N6-Methyladenosine modification in DNA (m6dA) is the most abundant DNA modification [28]. Recent research suggests that global cytosine methylation (5mC) patterns of genomic DNA (gDNA) from tumor tissues and cell-free DNA (cfDNA) from serum can be used as universal biomarkers for breast, prostate and colorectal cancers [29]. However, the global methylation pattern of 5mC, 5hmC and m6dA in salivary sEVs and whole saliva has not been assessed in relation to periodontal status.

The purpose of this pilot study was to investigate the global DNA epigenetic patterns, LPS-positive OMVs population and specific periodontal pathogen-derived OMVs in salivary sEVs from healthy, gingivitis and periodontitis patients and to determine the diagnostic power of aberrant DNA epigenetics or specific bacterial OMVs as potential biomarkers in periodontitis.

## 2. Results

### 2.1. Participant Characteristics

As shown in Table 1, the group of 22 participants (*n* = 22) was mixed gender (15 males, 7 females) and mostly non-smokers from various ethnic backgrounds (9 Caucasians and 13 Asians), with an age range from 24 to 66 years. It is noted that a statistically significant age difference was found between periodontitis and non-periodontitis patients.

### 2.2. Salivary sEV Characterisation and Quantification of CD9+ sEVs and LPS+ OMVs

**Figure 1:**
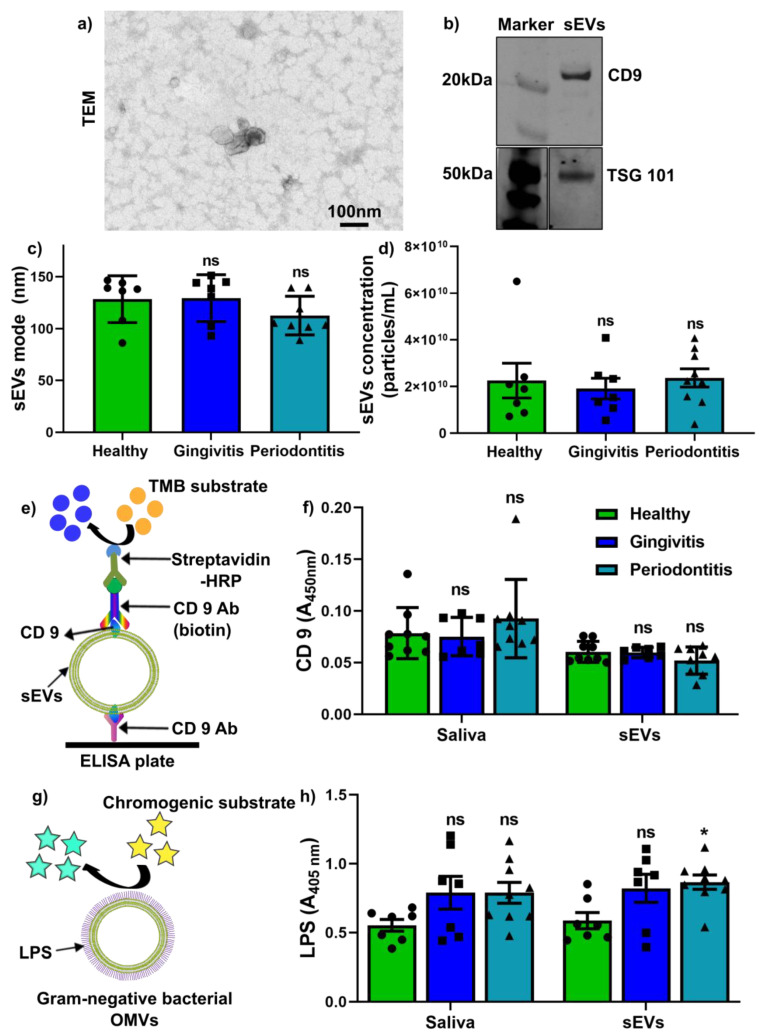
(**a**) A representative TEM image of isolated salivary small extracellular vesicles (sEVs). (**b**) Western blot analysis for TSG101 and CD9 for sEVs. (**c**,**d**) sEV average mode and particle concentration characterisation by nanoparticle tracking analysis (NTA). (**e**) Schematic of ELISA to measure CD9 concentration in whole saliva and purified sEVs. TMB: 3, 3′, 5, 5′-tetramethylbenzidine; streptavidin-HRP: streptavidin–horseradish peroxidase; Ab: antibody. (**f**) CD9 concentration measurement in the whole saliva and purified salivary sEVs. (**g**) Illustration of LPS + OMVs by an interaction between LPS and chromogenic substrates. LPS: lipopolysaccharide. (**h**) Levels of LPS in whole saliva and sEVs. *p* values were calculated vs. healthy groups. *: *p* < 0.05.

We isolated the salivary sEVs using the SEC method and verified them by transmission electron microscopy (TEM) and nanoparticle tracking analysis (NTA). Salivary sEVs were confirmed as having a cup-shaped morphology (Figure 1a), with sEV-associated protein (TSG101 and CD9) expression (Figure 1b). The NTA results demonstrated that the average mode (Figure 1c) and particle concentration (Figure 1d) of sEVs were comparable between the healthy, gingivitis and periodontitis groups.

We next investigated CD9 and LPS expression levels in saliva and salivary sEVs. Using our in-house CD9 enzyme-linked immunosorbent assay (ELISA, Figure 1e), we found that CD9 in saliva and CD9+ sEVs were comparable between the healthy, gingivitis and periodontitis groups (Figure 1f). Furthermore, an endotoxin quantification kit was used for LPS+ sEVs (Figure 1g) and the results showed that there was no statistically significant difference in saliva LPS in gingivitis and periodontitis groups compared to the healthy group, while LPS levels in sEVs were significantly increased in the periodontitis group when compared to healthy patients (Figure 1h).

### 2.3. Global Methylation Profile in Saliva and Salivary sEVs

**Figure 2:**
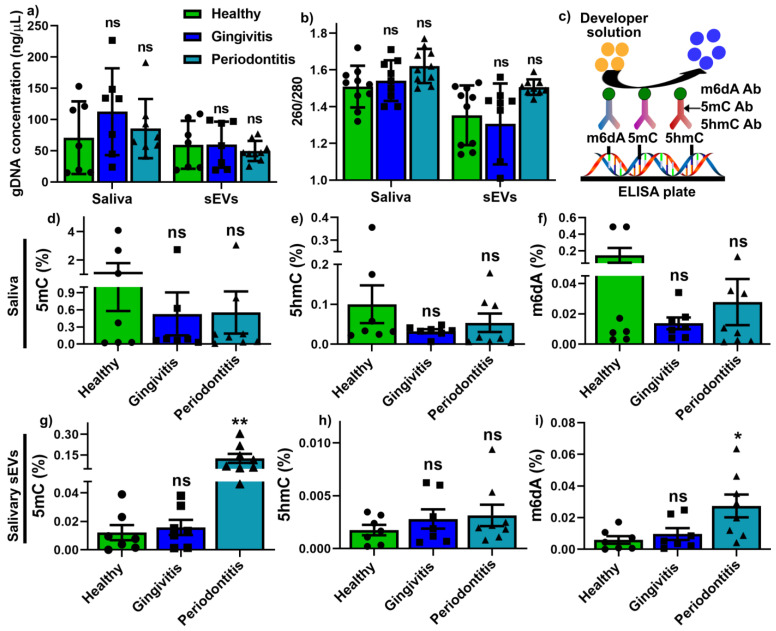
The genomic DNA (gDNA) concentration (**a**) and quality (**b**) from 200 μL saliva and 10 salivary sEV particles. (**c**) Schematic ELISA procedures for global DNA methylation (5mC), hydroxymethylation (5hmC) and m6A DNA methylation (m6dA) assays. (**d**–**i**) Quantification of global 5mC (**d**,**g**), 5hmC (**e**,**h**) and m6dA (**f**,**i**) contents in saliva and sEVs. *p* values were calculated vs. healthy groups. *: *p* < 0.05. **: *p* < 0.005.

The gDNA from 200 μL saliva and sEV-associated gDNA (from 10^9^ particles) were comparable in quantity (Figure 2a) and quality (Figure 2b) between healthy, gingivitis and periodontitis groups. Commercial Abcam global 5mC, 5hmC and m6dA ELISA kits were used to measure the global methylation for both saliva and sEV samples, as illustrated in Figure 2c. The results demonstrated that in saliva, there was no significant difference in global 5mC (Figure 2d), 5hmC (Figure 2e) and m6dA (Figure 2f) methylation between the healthy, gingivitis and periodontitis groups. However, periodontitis sEVs exhibited significantly increased global 5mC (Figure 2g) and m6dA (Figure 2i) methylation compared to those from the healthy groups, while there was no change regarding sEV h5mC methylation (Figure 2h) between the three groups.

### 2.4. Bacterium and OMV Detection in Saliva and Salivary sEVs

**Figure 3:**
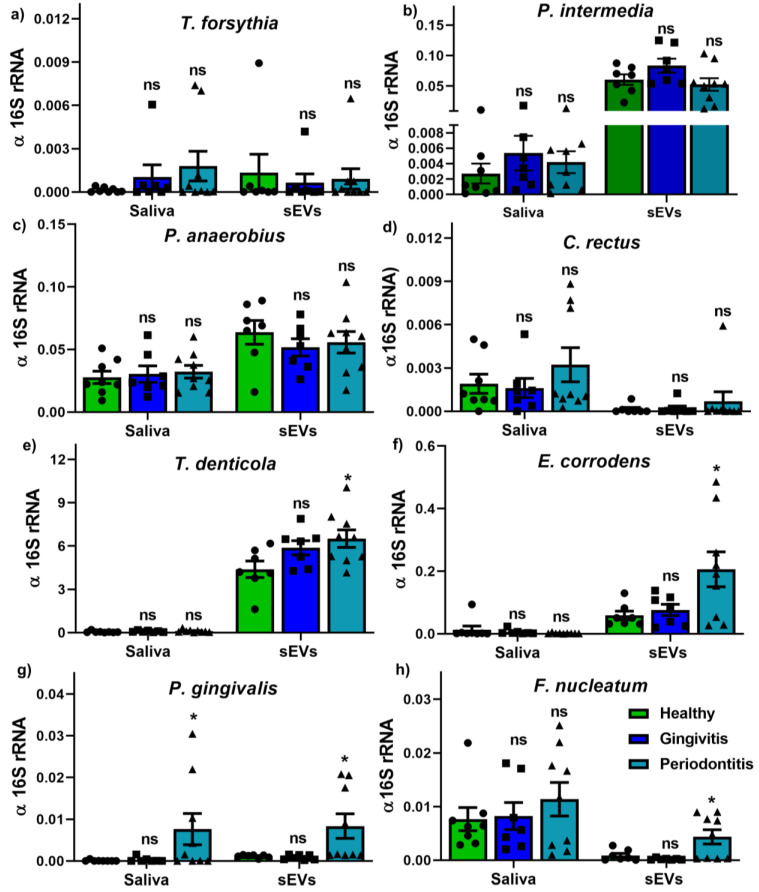
The detection of periodontal pathogens in saliva and salivary outer membrane vesicles (OMVs) within sEV population for eight Gram-negative putative periodontal pathogens (**a**–**h**). *p* values were calculated vs. healthy groups. *: *p* < 0.05.

Our pilot study identified the presence of periodontal pathogen-secreted outer membrane vesicles (OMVs) in isolated salivary sEVs using genomic DNA qPCR (Figure 3). The results demonstrated that, except for salivary *P. gingivalis* (Figure 3g), all the other bacterial strains (Figure 3a–h) showed no difference in saliva between healthy, gingivitis and periodontitis groups. Meanwhile, only periodontitis salivary sEVs contained higher levels of periodontal pathogens, including *T. denticola* (Figure 3e), *E. corrodens* (Figure 3f), *P. gingivalis* (Figure 3g) and *F. nucleatum* (Figure 3h).

### 2.5. Discrimination Power of Up-Regulated Parameters in Periodontitis

**Figure 4:**
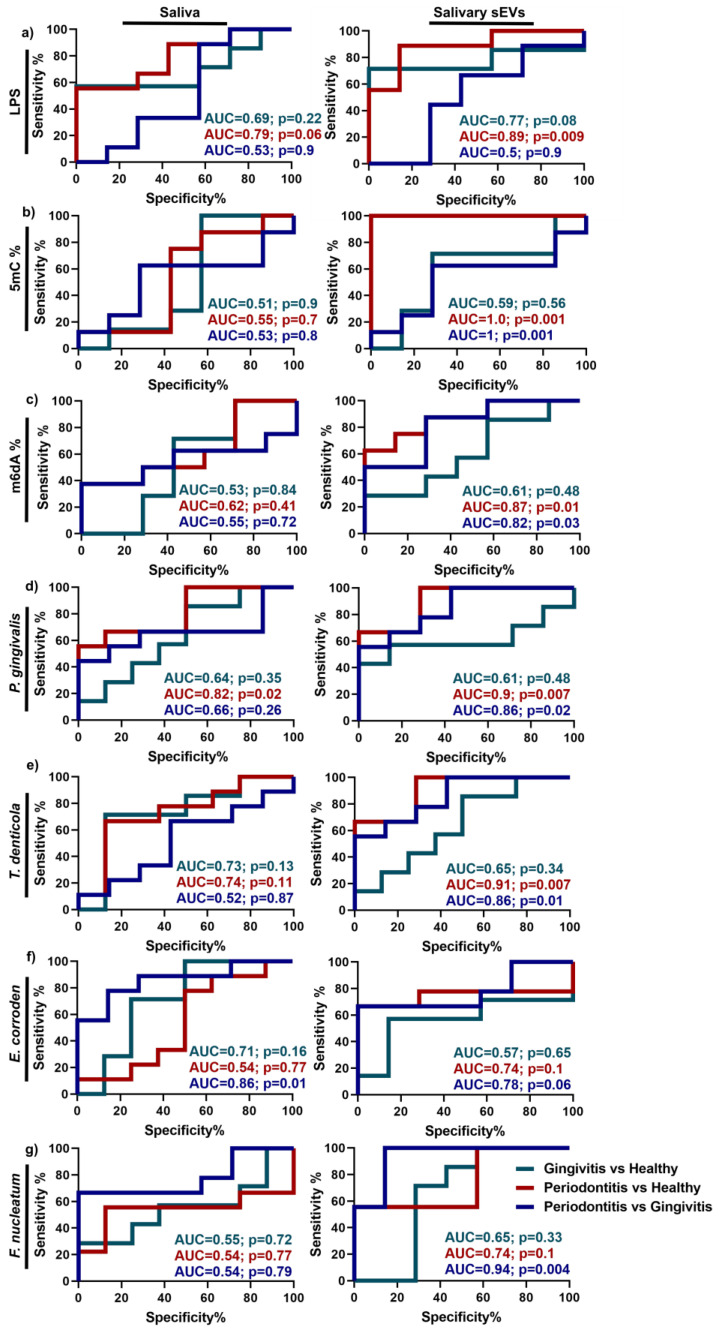
Discrimination power of up-regulated LPS (**a**), global 5mC (**b**), m6dA (**c**), *P. gingivalis* (**d**), *T. denticola* (**e**), *E. corrodens* (**f**) and *F. nucleatum* (**g**) in saliva and salivary sEVs from healthy, gingivitis and periodontitis patients by using receiver operating characteristic (ROC) curves and area under the curve (AUC).

Our research showed that periodontitis sEV LPS+ OMVs, global 5mC methylation and OMVs secreted by four periodontal pathogens (*T. denticola*, *E. corrodens*, *P. gingivalis* and *F. nucleatum*) were significantly increased compared to the healthy group. Consequently, receiver operating characteristic (ROC) curves and area under the curve (AUC) values were used to examine the discrimination power of these differentially regulated parameters as potential biomarkers for periodontal disease compared to healthy patients (Figure 4). The data showed that most of the parameters in salivary sEVs performed better in discriminating between gingivitis, periodontitis and healthy subjects compared to saliva (Figure 4a–g). Peridontitis sEVs had a far better discriminatory performance in the selected parameters than gingivitis sEVs when both were compared to healthy patients. Of note, LPS+ OMVs (Figure 4a), global 5mC methylation (Figure 4b), *P. gingivalis* OMVs (Figure 4d) and *T. denticola* OMVs (Figure 4e) in periodontitis sEVs showed high AUC values at 0.89, 1, 0.9 and 0.91, respectively, compared to healthy patients, whereas the corresponding gingivitis AUC values were 0.77 (LPS+ OMVs), 0.59 (global 5mC methylation), 0.61 *(P. gingivalis* OMVs) and 0.65 (*T. denticola* OMVs).

The discrimination power of tested parameters between gingivitis and periodontitis was also carried out. Except salivary *E. corroden* (AUC = 0.86), salivary EVs displayed a better performance in distinguishing gingivitis from periodontitis. It is noted that global 5mC methylation (AUC = 1) and *F. nucleatum* OMVs (AUC = 0.94) in salivary sEVs can distinguish gingivitis from periodontitis.

## 3. Discussion

This pilot study has provided fundamental insight into the human global DNA methylation profiles of sEVs and Gram-negative bacterial OMVs in different periodontal conditions (healthy, gingivitis and periodontitis). Our research showed that LPS + OMVs, global 5mC methylation and four periodontal pathogen (*T. denticola*, *E. corrodens*, *P. gingivalis* and *F. nucleatum*) OMVs were significantly increased in periodontitis sEVs compared to sEVs those from healthy groups. Notably, salivary sEV global DNA methylation (5mC) appears to be a highly sensitive marker (with an AUC of 1) for differentiating periodontitis and healthy patients. Conversely, in the whole saliva samples, only *P. gingivalis* showed a statistically significant increase (*p* < 0.05) in periodontitis compared to healthy patients, and the discriminatory power of this marker was low (AUC = 0.82). Generally, sEVs appear to have superior potential compared to whole saliva samples as diagnostic biomarkers of periodontitis. This may be because sEVs are more likely to reflect the local oral environment compared to the whole saliva, which reflects an individual’s overall systemic status. This is consistent with our findings that sEV miRNAs are better biomarkers for periodontitis than whole saliva [16].

Salivary sEV isolation methods are still being developed. Four published studies reporting on salivary sEV diagnosis research in periodontitis used either a precipitation-based ExoQuick kit [14,15,19] or ultracentrifuge method [20], with potential nuclear acid and protein contamination [30]. Our very recent research used the SEC method to isolate salivary sEVs [16,18] with enriched salivary sEV particles compared to the ultracentrifuge method [18], and this approach was applied in the current study. Our current study was consistent with our previous study [16], showing that salivary sEV mode and particle concentration were comparable between healthy, gingivitis and periodontitis groups.

Extracellular vesicles (EVs) are heterogeneous populations secreted by all species and play vital roles in physiologic and pathological cellular processes [31]. The role of various subpopulations of sEVs from different tetraspanin proteins (CD63+, CD9+, CD81+ subtypes) in periodontal disease has not been widely investigated and requires further elucidation. A recent report demonstrated salivary CD9+ and CD 81+ sEVs were decreased in periodontitis patients [14], while another report showed that salivary CD63+ sEVs are comparable in periodontitis and non-periodontitis patients [17]. In our study, we developed an in-house CD9 ELISA assay to quantify CD9+ sEVs, and the results demonstrated that salivary CD9 expression and salivary CD9+ sEVs were comparable between healthy, gingivitis and periodontitis groups, which was in line with Chaparro Padilla et al. [17].

It is widely recognised that certain Gram-negative periodontal pathogens are associated with periodontal diseases, such as *T. forsythia, P. gingivalis, T. denticola, P. intermedia, F. nucleatum, C. rectus, P. anaerobius* and *E. corrodens* [21,22]. Thus, detection of these bacteria in saliva has been used for periodontal disease diagnosis. In line with Damgaard et al. [32], our results demonstrated that salivary *P. gingivalis* is significantly increased in periodontitis patients compared to healthy patients.

Outer membrane vesicles (OMVs) are Gram-negative bacterial by-products, enriched with lipopolysaccharide (LPS) [23], which can be potentially used to quantify OMV subpopulations among isolated salivary sEVs. The data showed that salivary LPS was not statistically significantly different in the healthy, gingivitis and periodontitis groups, while LPS+ OMV populations were significantly enhanced in periodontitis patients compared to healthy patients. The diagnostic power of salivary LPS and LPS+ OMVs was also evaluated and LPS+ OMVs showed good biomarker discrimination (AUC = 0.89) for periodontitis, compared to the healthy group. To further confirm which bacterial strains contributed to the LPS+ OMVs, gDNA qPCR was performed for purified salivary sEVs. The data demonstrated that *P. gingivalis*-, *T. denticola*-, *E. corrodens*- and *F. nucleatum*-secreted OMVs were significantly enhanced in periodontitis patients compared to healthy patients. It was noted that *P. gingivalis and T. denticola* OMVs are good discriminators (AUC at 0.9 and 0.82, respectively), suggesting that they are strongly associated with periodontitis. Notwithstanding the limitation of the small sample size of this pilot study, our data provide initial evidence that LPS+ OMVs, *P. gingivalis* OMVs and *T. denticola* OMVs could be potential diagnostic biomarkers for periodontitis. However, this concept can only be fully explored once pure microbial OMVs or OMVs from a specific bacterial strain can be isolated from saliva and validated with a larger patient cohort.

As periodontal diseases are associated with genetic and epigenetic factors, it is important to understand salivary DNA epigenetics in different periodontal states. DNA methylation is an epigenetic mechanism involving the addition of a methyl group to cytosine or adenosine. 5-methylcytosine (5mC), 5-hydroxymethylcytosine (5hmC) and N6-methyladenosine modifications (m6dA) are the most critical DNA methylation epigenetics in regulating physiological and pathological processes [27,28]. It has been reported that increased global DNA methylation (5-methylcytosine, 5mC) is associated with diabetes and breast/prostate/colorectal cancers [29,33,34], however, there have been no attempts at investigating global 5mC, 5hmC and m6dA methylation levels in both saliva and salivary sEVs in periodontal disease. Moreover, in the periodontology field, most studies focus on gene-specific DNA methylation in gingival tissues or blood samples (reviewed in [6,7,8,35]). Our current pilot study is the first to utilise simple ELISA kits (turnaround time of approximately 2 h) to investigate global DNA epigenetics. While no differences in global methylation could be detected between the whole salivary samples from the three groups, significantly increased global 5mC and m6dA were detected in periodontitis sEVs compared to the healthy controls, with good discriminatory power (AUC = 1, 0.87, respectively). These differences in global methylation require further investigation in larger patient cohorts. Further, whole methylome investigations, such as using bisulphite sequencing or methylated DNA immunoprecipitation sequencing, could be used to determine individual salivary sEV methylation candidate biomarkers associated with different periodontal states.

Aside from the small sample size, a limitation of this pilot study was the age difference between the control/gingivitis and periodontitis groups, which should be addressed in future studies by recruiting older healthy and gingivitis patients. Another limitation of this study was that smoking status is different among the groups (two smokers in the periodontitis group; others are non-smokers). Thus, future studies should recruit a large cohort with age, gender and smoking status-matched non-smoker participants to confirm these findings.

In summary, notwithstanding the limitations of this pilot study, the findings suggest that sEVs may represent superior biosamples compared to the whole saliva for assessing periodontal status via global methylation and OMV assessment.

## 4. Materials and Methods

### 4.1. Participant Recruitment

This pilot study recruited 22 participants, with approved human ethics and written informed consent from the University of Queensland Human Ethics Committee (approval number 2018001225; approval date: 12 November 2018). Written informed consent was obtained from all subjects involved in the study with the following inclusion and exclusion criteria: (a) inclusion criteria: ≥18 years old; ≥20 teeth (excluding third molars); no periodontal treatment or antibiotic therapy three months prior to investigation, no long-term use of anti-inflammatory drugs; (b) exclusion criteria: metabolic bone diseases, autoimmune disease, unstable diabetes or post-menopausal osteoporosis, pregnancy. The Florida probe periodontal charting system (Florida Probe Corporation Gainesville, FL, USA) was calibrated and used to collect periodontal parameters using a standardised probing force. Comprehensive periodontal charting was performed by two independent experienced periodontists for each participant to determine bleeding on probing (BOP) and periodontal pocket depths (PPDs). Healthy (*n* = 7), gingivitis (*n* = 7) and periodontitis (*n* = 8) subjects were defined as described previously [16,18] and the new classification of periodontitis guidelines [2]: (a) healthy: no periodontal disease history; PPD < 3 mm; BOP < 15 % sites; (b) gingivitis: no periodontal pocket, PPD < 3 mm; BOP > 30 % sites; (c) stage III/IV periodontitis: > 30% of the sites with PPD ≥ 3 mm and BOP, at least five sites with PPD ≥ 5mm on at least three non-adjacent teeth. All participants had no underlying systemic diseases. The clinical characteristics of the participants are shown in Table 1.

Unstimulated whole saliva samples were collected before the full-mouth periodontal charting, as described previously [16,18,36], by asking the participants to spit the saliva samples into a sterile tube. The saliva samples were collected between 9 a.m. and 12 p.m. from the participants who refrained from eating and drinking for at least 1 h (up to 2 h). Before the saliva collection, the participants were asked to rinse their mouth to remove any food debris using 10 mL of water. The whole saliva was collected by spitting into a sterile Falcon tube, and the samples were kept on ice. Then, fresh saliva was aliquoted and frozen in a −80 °C freezer.

### 4.2. Salivary sEV Isolation and Characterisation

Salivary sEVs were isolated using size exclusion chromatography (SEC) columns (miniPURE-EVs, HansaBioMed, Lonza, Tallinn, Italy) according to the manufacturer’s instructions and as described previously [16,18]. Briefly, 250 μL of saliva were diluted in 250 μL of 1x phosphate-buffered saline (PBS, without calcium, magnesium, phenol red; In Vitro Technologies Pty Ltd., Australia). The samples were subjected to differential centrifugation at 4 °C: 300 g for 15 min, 2600 g for 15 min, 16,000 g for 20 min, prior to fractionating the supernatant on an SEC column. Each 100 μL fraction was collected; fractions 7 to 11 were obtained and concentrated to 100 μL using an Amicon Ultra 0.5 Centrifugal Filter Unit (10 kDa, Merck Millipore, QLD, Australia) by centrifugation at 14,000 g for 5 min at 4 °C.

Following the recommendation from the International Society of Extracellular Vesicles [37], sEVs were characterised via morphology by transmission electron microscopy (TEM), EV-associated protein markers by Western blot and sEV particle numbers by nanoparticle tracking analysis (NTA).

The TEM analysis was described previously [16,38]. Briefly, sEV samples were fixed in 3% glutaraldehyde and adsorbed on Formvar carbon-coated electron microscopy grids. After washing with PBS, the grids were placed in uranyl-oxalate solution (pH 7) for 3 min and then imaged using an FEI Tecan 12 transmission electron microscope (FEI, Hillsboro, OR, USA).

Western blotting was performed to detect sEV-associated proteins markers: CD 9 and tumour susceptibility gene 101 (TSG 101), as previously described [16,39,40,41,42,43]. Briefly, the sEV protein samples were separated by SDS-PAGE and transferred to a polyvinylidene difluoride membrane. The membrane was blocked with Odyssey^®^ Blocking Buffer at room temperature for 1 h, and primary antibodies (CD9, 1:1000, Santa Cruz Biotechnology; TSG101, 1:1000, Santa Cruz Biotechnology, Dallas, TX, USA) were incubated overnight at 4 °C. Then, the membrane was incubated with anti-rabbit DyLight 800 secondary antibody (1:10,000 in Odyssey Blocking Buffer) and anti-mouse DyLight 700 secondary antibodies, prior to being visualised on an Odyssey^®^ Infrared Imaging System (LI-COR Biotechnology, Inc., Lincoln, NE, USA).

The NTA analysis was carried out to determine the sEV particle size and concentration, as described previously [16]. Polystyrene latex beads (100 nm, Malvern NTA 4088) were used as a positive control and 1x PBS was used as a negative control. A NanoSight NS500 instrument (NanoSight, Salisbury, UK) with a 488 nm laser module and NTA 3.1 software was used to obtain 5 videos of 30 s for each sample with a camera level of 14 and a detection threshold set at 5. The video file was processed and analysed to determine the mode of particle size and particle concentration.

### 4.3. Determination of CD9 and Lipopolysaccharide (LPS) in Saliva and sEVs

To quantify saliva CD9 and CD9+ sEVs, an in-house enzyme-linked immunosorbent assay (ELISA) was developed following PeproTech’s TMB ELISA Development Kit protocol (illustrated in Figure 1e). For CD9 quantification, 12.5 μL of whole saliva and 10^8^ sEVs particles from each group were used. Briefly, the ELISA plates were coated with 4 μg/mL of monoclonal mouse anti-human CD9 antibody (HansaBioMed, Lonza) overnight at 4 °C. After 1 h of blocking, saliva or salivary sEVs samples were added and incubated for 1 h at 37 °C. After washing 3 times with 1% Tween 20 in PBS, monoclonal biotin-conjugated mouse anti-human CD9 at 4 μg/mL (HansaBioMed, Lonza) was added to the wells to bind with sEVs or saliva. After 3 further washes with PBST, diluted streptavidin–HRP conjugated HRP (1:2000) was added and incubated for 30 min at 37 °C. Then, 3,3′,5,5′-Tetramethylbenzidine (TMB) substrate was used for colour development and stopped with 1 M HCl stop solution. The OD value was be measured at 450 nm.

According to the manufacturer’s protocol, a Pierce^TM^ Chromogenic Endotoxin Quantification Kit (ThermoFisher Scientific) was used to determine endotoxin LPS levels by measuring the interaction of endotoxin (LPS) with proenzyme Factor C. For LPS quantification, 12.5 μL of whole saliva and 10^8^ sEV particles from each group were used after measuring the yellow colour at an absorbance of 405 nm (illustrated in Figure 1g).

### 4.4. Genomic DNA Isolation and DNA Methylation Landscape Quantification

Genomic DNA (gDNA) was isolated from 200 μL of saliva and 10^8^ sEV particles, using Trizol^TM^ reagent (Invitrogen, ThermoFisher Scientific, Melbourne, Australia) following the manufacturer’s instructions and as described previously [36]. The DNA quality and quantity were measured using a NanoDrop™ One spectrophotometer (Thermo Fisher Scientific, Australia).

Global methylation analysis of 5-methylcytosine (5mC), 5-hydroxymethylcytosine (5hmC) and N6-methyladenosine (m6dA) for DNA was performed by using a Global DNA Methylation Assay Kit (5mC, ab233486, Abcam), a Global DNA Hydroxymethylation Assay Kit (5hmc, ab233487, Abcam) and an m6dA DNA Methylation Assay Kit (m6dA, ab233488, Abcam), respectively, as per the manufacturer’s instructions. Briefly, sample DNA, positive controls at 6 different concentrations (to generate standard curves) and the negative control were mixed with DNA-binding solution and incubated at 37 °C for 60 min. After washing three times with 150 μL washing buffer, 5mC/5hmC/m6dA antibodies along with signal indicator and enhancer solution were added and incubated at room temperature for 1 h. Following thorough washing (five times) with wash buffer, 50μL developer solution were added and incubated for 3 min at room temperature until the positive control with the highest concentration turned blue. Subsequently, 50 μL of stop solution were added to each well for 2 min to stop the enzyme reaction. The absorbance was measured at 450 nm for 2 min on an Infinite Pro spectrometer.

The global methylation level of all DNAs was calculated using the following equations:

5mc/5hmC %=∗100%

m6dA %=∗100%

where the slope (OD/1%) was determined from the standard curve using linear regression; S = amount of input sample DNA in ng; P = amount of input positive control in ng; OD = optical density.

### 4.5. gDNA Real-Time Quantitative PCR (qPCR)

Salivary bacterial outer membrane vesicles (OMVs) and bacteria were detected using gDNA qPCR, as described previously [36]. The primers for 8 periodontal pathogens (*Tannerella forsythia, Porphyromonas gingivalis, Treponema denticola, Prevotella intermedia, Fusobacterium nucleatum, Campylobacter rectus, Peptostreptococcus anaerobius* and *Eikenella corrodens*) are listed in Table 2.

The gDNA qPCR was performed using SYBR Green reagent in StepOnePlus equipment (Applied Biosystems). The relative expression was normalised with 16S rRNA and calculated as 2 ^-(normalised average Cts)^.

### 4.6. Statistical Analysis

All the data are presented as mean ± standard deviation (SD) and ordinary one-way ANOVA with Tukey’s multiple comparison test (a single pooled variance) was used to compare the data between healthy, gingivitis and periodontitis patients with GraphPad Prism 8.3.1 software (San Diego, SA, USA). The normality of data distribution was calculated in Prism using a QQ plot. *p* < 0.05 was considered a statistically significant difference.

Receiver operating characteristic (ROC, using the Wilson/Brown method) curves and the area under the curve (AUC) were generated using data from healthy controls and patients (gingivitis and periodontitis, respectively) with GraphPad Prism 8.3.1 software (San Diego, SA, USA). The area under the curve (AUC, indicating the discriminatory power of the biomarkers) and *p* values were calculated by the software.

## Figures and Tables

**Table 1:** Participants’ characteristics of the study.

		Healthy (*n* = 7)	Gingivitis (*n* = 7)	Periodontitis (*n* = 8)
**Gender**	Male	4 (57.1%)	6 (85.7%)	5 (62.5%)
	Female	3 (42.9%)	1 (14.3%)	3 (37.5%)
**Age**		35.1 ± 7.8 (24–48)	30.5 ± 5.7 (28–40) *p* = 0.57	48.2 ± 10.6 (38–66) * *p* = 0.02
**Smoking habit**	Non-smokers	7 (100%)	7 (100%)	6 (75%)
	Smokers	0	0	2 (25%)
**Ethnicity**	Caucasians	4 (57.1%)	2 (28.5%)	3 (37.5%)
	Asians	3 (42.9%)	5 (71.5%)	5 (62.5%)
**BOP**		11% ± 3.1 (6%–14%)	48.5 % ± 14.6 (34%–70%) * *p* = 0.0001	47.5 % ± 18 (25%–74%) * *p* = 0.0001
**PI**		15.1 % ± 6.9 (6%–28%)	51.5 % ± 27 (9%–89%) * *p* = 0.08	77.3 % ± 16.7 (53%–94%) * *p* = 0.0006
**No. of deep pockets** (**≥5 mm**)				24 ± 21.4 (5–55)
**Average PPD** (**mm**)				4.95 ± 1.4 (2.5–6.4)
**Periodontitis** **classification**	Localised (<30%)			3 (37.5%)
	Generalised (≥30%)			5 (62.5%)
	Grade B			5 (62.5%)
	Grade C			3 (37.5%)
	Stage III			7 (85.7%)
	Stage IV			1 (14.3%)

Note: *p* values were calculated versus healthy controls. *: *p* < 0.05.

**Table 2:** The primers used in this study.

	Forward Primer (5′-3′)	Reverse Primer (5′-3′)
*T. forsythia*	GGGTGAGTAACGCGTATGTAACCT	CCCATCCGCAACCAATAAA
*P. gingivalis*	TGCAACTTGCCTTACAGAGGG	ACTCGTATCGCCCGTTATTC
*T. denticola*	TGGTGAGTAACGCGTGGGTGACCT	TTCACCCTCTCAGGCCGGA
*P. intermedia*	CCACATATGGCATCTGACGTG	CACGCTACTTGGCTGGTTCA
*F. nucleatum*	GGATTTATTGGGCGTAAAGC	GGCATTCCTACAAATATCTACGAA
*C. rectus*	TTTCGGAGCGTAAACTCCTTTTC	TGATTCCGAGTAACGCTTGCA
*P. anaerobius*	GGGTGAGTAACGCGTGGGT	TACTGATCGTCGCCTTGGTGG
*E. corrodens*	ACGTCCTACGGGAGAAAGCGG	CCATTGTCCAAAATTCCCCACTG
*16S rRNA*	TGGAGCATGTGGTTTAATTCGA	TGCGGGACTTAACCCAACA

## Data Availability

The data that support the findings of this study are available from the corresponding author upon reasonable request.
